# Deaths and Medical Visits Attributable to Environmental Pollution in the United Arab Emirates

**DOI:** 10.1371/journal.pone.0057536

**Published:** 2013-03-04

**Authors:** Jacqueline MacDonald Gibson, Jens Thomsen, Frederic Launay, Elizabeth Harder, Nicholas DeFelice

**Affiliations:** 1 Department of Environmental Sciences and Engineering, Gillings School of Global Public Health, University of North Carolina–Chapel Hill, Chapel Hill, North Carolina, United States of America; 2 Health Authority–Abu Dhabi, Abu Dhabi, United Arab Emirates; 3 Environment Agency–Abu Dhabi, Abu Dhabi, United Arab Emirates; Tehran University of Medical Sciences, Iran (Republic of Islamic)

## Abstract

**Background:**

This study estimates the potential health gains achievable in the United Arab Emirates (UAE) with improved controls on environmental pollution. The UAE is an emerging economy in which population health risks have shifted rapidly from infectious diseases to chronic conditions observed in developed nations. The UAE government commissioned this work as part of an environmental health strategic planning project intended to address this shift in the nature of the country’s disease burden.

**Methods and Findings:**

We assessed the burden of disease attributable to six environmental exposure routes outdoor air, indoor air, drinking water, coastal water, occupational environments, and climate change. For every exposure route, we integrated UAE environmental monitoring and public health data in a spatially resolved Monte Carlo simulation model to estimate the annual disease burden attributable to selected pollutants. The assessment included the entire UAE population (4.5 million for the year of analysis). The study found that outdoor air pollution was the leading contributor to mortality, with 651 attributable deaths (95% confidence interval [CI] 143–1,440), or 7.3% of all deaths. Indoor air pollution and occupational exposures were the second and third leading contributors to mortality, with 153 (95% CI 85–216) and 46 attributable deaths (95% CI 26–72), respectively. The leading contributor to health-care facility visits was drinking water pollution, to which 46,600 (95% CI 15,300–61,400) health-care facility visits were attributed (about 15% of the visits for all the diseases considered in this study). Major study limitations included (1) a lack of information needed to translate health-care facility visits to quality-adjusted-life-year estimates and (2) insufficient spatial coverage of environmental data.

**Conclusions:**

Based on international comparisons, the UAE’s environmental disease burden is low for all factors except outdoor air pollution. From a public health perspective, reducing pollutant emissions to outdoor air should be a high priority for the UAE’s environmental agencies.

## Introduction

Over the past half century, the United Arab Emirates (UAE) has developed at an unprecedented rate. Prior to the discovery of oil in 1958, the UAE (then called the Trucial States) was among the Arab world’s poorest nations, with “no electrical grid, indoor plumbing, telephone system, public hospital, or modern school” [1]. Fueled by oil, the UAE has transformed over the past 50 years. Steel-and-glass metropolises tower above the once barren desert, state-of-the-art desalination plants supply fresh water, factories export locally produced goods worldwide, and once-remote airports serve as international hubs. By 2010, the UAE boasted the world’s third-largest gross national income per capita: $59,993, trailing only Liechtenstein and Qatar [2].

Overall, development has dramatically improved public health in the UAE. Life expectancy has increased from less than 45 in the 1950s to 75.9 in 2010, and infant mortality rates have plummeted from more than 180 to fewer than 5 per 1,000 births [3]. However, as in every other nation undergoing an industrial transition, development also has created new forms of environmental pollution, leading to new health risks.

As part of an anticipatory project to confront emerging environmental risks and prevent downstream public health impacts, the Environment Agency–Abu Dhabi (EAD) commissioned a study to quantify the UAE’s environmental burden of disease (EBD). The EAD is charged with protecting environmental quality in Abu Dhabi, the UAE’s largest emirate (comprising 80% of the UAE’s land area and 33% of its population) and source of most of the UAE’s petroleum wealth [4]. The EAD collaborated with the Health Authority–Abu Dhabi (HAAD), responsible for monitoring and improving public health and regulating the health-care sector in Abu Dhabi emirate. The EAD and HAAD recruited an international research team of environmental and public health specialists to carry out the analysis. This article reports on the resulting UAE EBD estimates.

The World Health Organization (WHO) in 2007 released preliminary EBD estimates for nations around the world [5]. WHO’s EBD estimates included risks of four categories of environmental pollution: (1) particulate matter in outdoor air, (2) combustion of solid fuels indoors, (3) particulate matter and carcinogens in occupational environments, and (4) pathogens in water. The WHO attributed 13 million deaths each year to these risks, with the magnitude and relative importance of the risks differing considerably by epidemiologic subregion [6]. As a result of the high regional variability, a June 2007 editorial in *The Lancet* noted, “With its release of each country’s profile of environmental factors and their impact on health. WHO has made a first, very important step towards facilitating more joined-up thinking by policymakers when planning interventions that have the greatest effect at a population level,” but that these estimates “should only serve as a starting point for countries to collect their own data for refinement and validation” [6]. To our knowledge, the UAE is the first country to follow through on completing its own comprehensive EBD assessment, considering all the risk factors included in the WHO estimates and several others as well. As such, this project may serve as a model for similar projects in other countries.

Our research team constructed a computer simulation model, the *United Arab Emirates Environmental Burden of Disease Model*, to link data on environmental pollutant concentrations with new UAE public health data and with epidemiologic studies that estimate the relative risks of various illnesses due to pollutant exposures. To our knowledge, this model is the first to implement a comprehensive, national-scale EBD analysis in a flexible computer simulation platform that reflects uncertainty in the estimates and that can be readily updated in the future as conditions change and new local data are collected. The model is designed to support policy analyses comparing the effectiveness of alternative options for reducing environmental risks to health.

Although “environmental risk” may be defined much more broadly, for this project we focused on environmental risks that are within the mandate and capability of the EAD and HAAD to address. Specifically, we considered six categories of risk, corresponding to six different exposure routes:

1. outdoor air pollution,
2. indoor air pollution,
3. occupational exposures,
4. drinking water contamination,
5. coastal water pollution, and
6. global climate change.

This list of risk factors includes all those in the WHO preliminary estimates except for indoor air pollution due to solid fuel use, since solid fuel is no longer used for cooking in the UAE. We also included pollutants for each exposure route that were relevant to the UAE but were not considered in the previous WHO estimates (see “Pollutants,” below). Since the scope of potential pollutant-exposure route combinations is in the thousands, we narrowed the list through a two-step process. First, we conducted preliminary risk assessments for candidate pollutant-exposure route combinations identified by the EAD and the WHO Centre for Environmental Health Activities for the Eastern Mediterranean Region. Then, we presented the preliminary risk assessment results at workshops involving government environment and health officials, faculty at local universities, international experts, local industries, and environmental groups. Participants were led through a systematic process, developed through previous research, to prioritize the pollutant-exposure route combinations for consideration [7]. Stakeholder engagement in the selection of risks to consider was essential because the results were intended to inform future UAE strategic planning [8].

## Methods

### Study Population

The EBD estimates include all residents of the UAE’s seven emirates, including citizens and expatriates, during the year 2008 (Figure S1 and Table S1). Expatriates constituted 81% of the population–a result of workers emigrating to the UAE to fill jobs arising from the nation’s ambitious development agenda, which requires more manpower than is available from the indigenous population (864,000 in 2008). About 85% of the expatriates are Asians (mostly from India and Pakistan), and another 10% are Arab [9]. The remaining 5% includes Australasians, Europeans, and North Americans.

### Health Outcomes

We provide EBD estimates for two health outcome categories: mortality and morbidity, with the latter expressed as number of health-care-facility visits. Health-care facility visits include all patient use of hospitals, doctor’s offices, and pharmacies. Table 1 lists the causes of mortality, and Table 2 the illnesses considered in this research, along with the total numbers of each in 2008.

**Table 1:** Causes of mortality considered in this study.

Exposureroute	Cause of mortality	ICD-10 code(s)	Baseline mortality (deathsin 2008)	Pollutants	Exposure estimation method	Relative risk (95% CI)
Outdoor air pollution	All causes (adults>30)	N/A	8,865	PM_2.5_ (average annual concentration, µg/m^3^)	Abu Dhabi outdoor air quality monitors [14]	1.06 (1.02–1.11) (per 10 µg/m^3^); see [25]
	Respiratory disease(children <5)	J00–99	27	PM_10_ (average daily concentration, µg/m^3^)	Same as for PM_2.5_	1.017 (1.0034, 1.03) (per 10 µg/m^3^); see [26]
Indoor air pollution	Cardiovascular disease	I00–79	2,310	Environmental tobacco smoke (ETS), present or absent in home	Household surveys: ETS present in 19%of homes	Male nonsmokers: 1.25 (1.06, 1.47); female nonsmokers: 1.35 (1.11, 1.64) [27]
	Lung cancer	C33–4	120	ETS	Same as previous	Male nonsmokers: 1.1 (0.6, 1.8); female nonsmokers: (1.2 (0.8, 1.6) [28]
				Radon (average daily concentration, Bq/m^3^)	Household measurements* (Abu Dhabi Cityand Sharjah only): Abu Dhabi, lognormal(mean = 14.4, sd = 7.37); Sharjah, triangular(8, 50.3, 164); assumed zero elsewhere	1.08 (1.13, 1.16) (per 100 Bq/m^3^) [29]
				Incense use (frequency per week)	Household surveys: Daily users = 43.54% ofpopulation; intermittent users = 42.86%	Daily users: 1.8 (1.2, 2.6); intermittent users (1–5 times/week): 1.2 (0.9–1.6) [30]
Occupational exposures	Asthma	J45	10	Employment in occupation involving exposure to dusts, fumes	UAE Ministry of Economy data on workforceparticipation by industry sector andoccupation within sector; see [20]	Varies by occupation and gender; see [20]
	Chronic obstructive pulmonary disease	J44	37	Employment in occupation involving exposure to dusts, fumes	Same as previous	Varies by exposure level and gender; see [20]
	Asbestosis	501	0	Asbestos exposure	NA	100% of observed cases
	Malignant mesothelioma	C45	6	Asbestos exposure	NA	90% of observed cases in males and 25% in females; see [20]
	Silicosis	502	0	Silica exposure	NA	100% of observed cases
	Leukemia	C91–5	130	Employment in occupation with exposureto diesel exhaust, benzene, ethylene oxide	UAE Ministry of Economy data on workforceemployed by industry sector; CarcinogenExposure (CAREX) database; see [20]	Low exposure: 1.9 (1.6, 2.2); high exposure: 4 (3.6, 4.4); see [20]
	Lung cancer	C33–4	120	Employment in occupation with exposure to arsenic, asbestos, beryllium, cadmium, chromium, nickel, silica	Same as previous	Low exposure: 1.21 (1.18, 1.24); high exposure: 1.77 (1.71,1.83); see [20]
Climate change	Cardiovascular disease	I00–79	2,310	Increase in ambient temperature attributable to global climate change	100% of population exposed	1.001 (1.000, 1.003) [31]
Drinking water contamination	Bladder cancer	C67, C68	23	Drinking chlorinatedwater	Citizens: 10.5% consume tap (chlorinated)water; non-citizens: tap water consumptionrepresented as uniform (84%, 96.4%)distribution**	Males: 1.24 (0.97, 1.57); females: 1.17 (1.03, 1.34) [32]
	Colon cancer	C18	80	Same as previous	Same as previous	Males: 1.09 (0.81, 1.48); females: 1.19 (0.93, 1.53) [32]
	Rectal cancer	C19–21	30	Same as previous	Same as previous	Males: 1.24 (0.86, 1.79); females: 1.10 (0.90, 1.36) [32]
	Gastroenteritis	A00–9	7	Access to regulated drinking watersupply and sewagetreatment	Population divided into two groups: (1) accessto regulated water supply and sanitation(population fraction represented as triangular (0.96, 0.98, 1.0) distribution); (2) access to improved but unregulated water, no sanitation	Group 1: uniform (1, 4); group 2: uniform (7.2, 10.2) [21], [33]

* EAD provided measured radon concentrations from 111 Abu Dhabi residential dwellings (202 measurements in total) and a mean, minimum, and maximum value for measurements taken in Sharjah.

** Our survey of 628 citizen households found 10.5% drink tap water, 84.6% bottled water, 3.4% well water, and 1.5% water from undefined other sources [19]. Estimates using bottled water industry sales data suggest noncitizens consume 84%–96.4% of water from taps [34], [35].

**Table 2:** Nonfatal illnesses considered in this study.

Exposureroute	Illness	ICD-9 code(s)	2008 health-care visits	Pollutants	Exposure estimation method	Relative risk
Outdoor air pollution	Cardiovascular disease	390–448	307,667	PM_10_ (daily average,µg/m^3^)	Abu Dhabi outdoor air qualitymonitors [14]	1.003 (1.0024–1.0036) (per 10 µg/m^3^); see [14]
	Respiratorydiseases	480–6; 490–7; 507	176,048	PM_10_ (daily average,µg/m^3^)	Same as previous	1.008 (1.0047–1.012) (per 10 µg/m^3^); see [14]
				Ozone (dailyaverage, ppb)	Same as for PM_10_	1.03 (1.02–1.05) (per 10 ppb); see [14]
Indoor airpollution	Asthma(age <18)	493	24,418	Mold (presencein home)	Household surveys: present in16% of homes [19]	1.35 (1.20, 1.51) [36]
				Environmental tobacco smoke (ETS) in home	Household surveys: present in 19% of homes [19]	1.48 (1.32, 1.65) [37]
	Asthma (age≥18)	493	32,388	Mold (presence in home)	Household surveys: present in 16% of homes [19]	1.54 (1.01, 2.32) [38]
	Asthma (age≤6)	493	13,879	Formaldehyde (daily average, µg/m^3^)	Household surveys: lognormal (mean = 22.5, sd = 63.6) [19]	1.003 (1.002, 1.004) (per 10 µg/m^3^) [39]
	Cardiovascular disease	390–448	307,667	ETS	Household surveys: present in 19% of homes [19]	1.25 (1.17, 1.32) [40]
	Lower respiratory tract infection(age≤ 6)	480–92	13,996	ETS	Same as previous	1.57 (1.28, 1.91) [41]
	Leukemia	204–208.9	1,520	ETS	Same as previous	2.28 (1.15, 4.53) [42]
	Lung cancer	162	444	Radon concentration (Bq/m^3^)	Data from Abu Dhabi Food Control Authority: Abu Dhabi City, lognormal (mean = 14.4, sd = 7.37); Sharjah, triangular (8, 50.3, 164); other emirates = 0	1.08 (1.13, 1.16) (per 100 Bq/m^3^) [29]
				Incense use (frequency)	Household surveys: Daily users = 43.54% of population; intermittent users = 42.86% [19]	Daily users: 1.8 (1.2, 2.6); intermittent users (1–5 times/week): 1.2 (0.9–1.6) [30]
Occupationalexposures	Asthma	493	72,301	Employment in occupation with dusts, fumes	UAE Ministry of Economy data on workforce participation by industry sector and occupation within sector [20]	Varies by occupation and gender; see [20]
	Chronic obstructive pulmonary disease	490–2, 494, 496	27,212	Same as previous	Same as previous	Varies by occupation and gender; see [20]
	Leukemia	204–208.9	1,520	Employment in occupation with diesel exhaust, benzene, ethylene oxide	UAE Ministry of Economy data on workforce employed by industry sector; Carcinogen Exposure (CAREX) database; see [20]	Low exposure: 1.9 (1.6, 2.2); high exposure: 4 (3.6, 4.4) [20]
	Lung cancer	162	443	Employed in occupation with arsenic, asbestos, beryllium, cadmium, chromium, nickel, silica	Same as previous	Low exposure: 1.21 (1.18, 1.24); high exposure: 1.77 (1.71,1.83); see [20]
	Malignant mesothelioma	163	28	Diagnosed mesothelioma	NA	90% of observed cases in males; 25% of cases in females; see [20]
	Asbestosis	501	3	Diagnosed asbestosis	NA	100% of observed cases
	Silicosis	502	8	Diagnosed silicosis	NA	100% of observed cases
Climatechange	Cardiovascular disease	390–448	307,667	Increase in annual average ambient temperature	100% of population exposed	1.001 (1.000, 1.003) [31]
Drinkingwatercontamination	Bladder cancer	188	929	Total trihalomethane (TTHM) concentration (µg/l)	10.5% of citizens consume tap water; noncitizen consumption represented as uniform (84%, 96.4%) distribution*	Males: 1.24 (0.97, 1.57); females: 1.17 (1.03, 1.34) [32]
	Colon cancer	153	2,191	TTHM (µg/l)	Same as previous	Males: 1.09 (0.81, 1.48); females: 1.19 (0.93, 1.53) [32]
	Rectal cancer	154	639	TTHM at tap (µg/l)	Same as previous	Males: 1.24 (0.86, 1.79); females: 1.10 (0.90, 1.36) [32]
	Gastroenteritis	008–9, 558.9	81,110	Availability of regulated drinking water supply and sewage treatment	Population divided into groups: (1) access to regulated water supply and sanitation (population fraction represented as triangular (0.96, 0.98, 1.0) distribution); (2) access to improved but unregulated water, no sanitation	Group 1: uniform (1, 4)Group 2: uniform (7.2, 10.2) [21]
Coastal water pollution	Gastroenteritis^4^	008–9, 558.9	81,110	Enterococci concentration in beach water (number/100 ml)	Water quality samples from Abu Dhabi beaches**; previous surveys of swimming frequency***	1.34 (1.00, 1.75) (per log-10) [43]

* Our survey of 628 citizen households found 10.5% drink tap water; 84.6% bottled water; 3.4% well water; and 1.5% water from undefined other sources [19]. Estimates using bottled water industry sales data suggest noncitizens consume 84%–96.4% of water from taps [34], [35].

** Monthly observations were available for two Abu Dhabi beaches for 2006. We therefore represented coastal water quality in each month as a uniform distribution with a lower bound equal to the lowest observed concentration and an upper bound equal to the highest observed concentration. Due to wastewater overflows in Dubai over the time period of this study, we assumed enterococci concentrations at Dubai beaches were twice those observed in Abu Dhabi (while for all other emirates, concentrations were assumed the same as in Abu Dhabi). The uniform distribution parameters for all emirates other than Dubai (in enterococci/100 ml) are as follows: Jan. (2, 8); Feb. (0, 4); Mar (0, 3); Apr (0, 0); May (0, 0); Jun (0, 12); Jul (0, 85); Aug (0, 85); Sept. (0, 43); Oct. (4, 250); Nov. (5, 6); Dec. (3, 12). For Dubai, these parameters were doubled.

*** Proportion of citizens swimming in coastal waters in any given month were estimated from Badrinath et al. [44], as follows: males ≤14: 3.8%, males >14, 1.4%; females ≤14, 0.87%; females >14, 0%. Proportion of non-citizens swimming in coastal waters were estimated from the Australian Sports Commission [45], assumed to be 6.2% (both genders, all ages).

To support this analysis, HAAD compiled death records for Abu Dhabi emirate for 2008. The records included 2,949 deaths listed by cause (by ICD-10 code), time, location, age, gender, and nationality. This database includes all deaths reported in Abu Dhabi for the study year. HAAD considers the death notification rate to be 100%, since it is not legal to bury, cremate, or expatriate a body without a death certificate. Hence, death rates estimated from this data set should be very accurate. Comparable information was not available from other emirates. Baseline death information for the other emirates was estimated by calculating death rates for gender-citizenship groups in Abu Dhabi and then applying those rates to population estimates for those same demographic groups in the other emirates.

HAAD also provided patient encounter records for the diseases of interest in this study from Abu Dhabi’s largest health insurance provider, Daman, which covers 73% of the emirate’s population. We used these data as a surrogate for morbidity estimates because they provided the most accurate and comprehensive database of incidences of illness available for this research. Prior to 2008, Abu Dhabi lacked standards for medical records coding; hence, the data set employed in this research is the first in Abu Dhabi to be compiled and quality-assured according to international best practices in medical records management [10]. The records included the date of encounter, ICD-9 code for the corresponding illness, health-care facility name, and patient demographic information (age, gender, citizenship). Patient identifiers were not provided. Noninfectious disease health-care facility visit records were provided for 2008 (162,228 visits recorded by Daman). Gastrointestinal illness records (10,581 in total) were provided for the first half of 2009; we assumed this represented 50% of the visits that would have occurred in 2008 among individuals covered by Daman. Like the mortality data, the health insurance claims data were scaled to cover the entire UAE population.

It is possible that using the Daman data as the basis for estimating health-care facility visit rates may have biased our results because the population insured by Daman may not be representative of the UAE population as a whole. Health insurance is mandatory in Abu Dhabi, and Daman is the major insurance provider, covering all Emiratis, all unskilled expatriate laborers, and many higher skilled expatriates. However, some highly skilled expatriates purchase enhanced insurance from one of dozens of private companies [11]. Hence, it is possible that the Daman data set under-represents skilled expatriate workers. These skilled workers may be healthier than unskilled workers due to their higher socioeconomic status. However, previous analyses have shown that skilled workers visit health-care facilities more often than unskilled workers (3.8 versus 2.7 visits per subscriber per year, respectively) [10]. Hence, the direction of the bias introduced by under-representing skilled workers is unknown, since on the one hand excluding wealthier workers would be expected to bias predicted disease rates upwards while on the other hand wealthier workers visit doctors more often than their lower-wage counterparts, creating the potential for downward bias by excluding these workers. Nonetheless, data from the other insurance providers, which are private, were not available to support this analysis, and the HAAD data from Daman represent the most comprehensive and most effectively quality-assured health data available for our study year [10].

### Pollutants

As in previous EBD studies (see Table S2 for a listing), we focused on pollutants for which exposure is potentially common and strong epidemiologic evidence is available to predict the occurrence of illness due to contaminant exposure. As noted above, preliminary risk assessments by experts and stakeholder engagement sessions narrowed the list of candidates [7]. Tables 1 and 2 list the pollutants for each exposure route-health endpoint combination. In the case of radon, exposure data were available only for a subset of the population (residents of the city of Abu Dhabi and the emirate of Sharjah), so we report those results separately.

### EBD Estimation Method

To estimate the burden of disease due to each combination of exposure pathway and pollutant, we used the “population attributable fraction” (*PAF*) approach. The *PAF* is the proportion of reduction in disease or mortality that would be expected if exposure to a pollutant were reduced to an alternative (known as “counterfactual”) level and can be computed from equation 1 [12], [13].
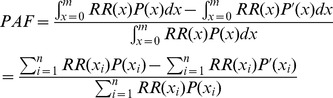
(1)where *x* is the exposure level; *RR(x)* is the relative risk at exposure level *x*; *P(x)* is the population distribution of exposure; *P’(x)* is the alternative or counterfactual distribution of exposure; *m* is the maximum possible exposure level; and n is some finite number of discrete exposure intervals. For this study, the counterfactual exposure level was the elimination of all pollutants to background levels. Background levels were assumed equal to zero for all exposure pathways except for outdoor air, for which background concentrations were represented as uniform distributions with parameters (10, 90) µg/m^3^, (5, 35) µg/m^3^, and (0, 25) ppb for PM_10_, PM_2.5_, and O_3_, respectively, where the first parameter represents the lower bound and the second the upper bound of the distribution [14]. We estimated the EBD separately for each exposure route (i.e., we assumed all other exposure routes were unchanged while exposures via the given route were decreased). For the indoor air exposure route, mold, environmental tobacco smoke, and formaldehyde in indoor air all can exacerbate asthma in children; therefore, we estimated the total *PAF* for that exposure route from equation 2 [13].
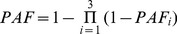
(2)where *PAF_i_* is the *PAF* for pollutant i (mold, environmental tobacco smoke, or formaldehyde). Equation 2 assumes that the exposures to the three pollutants are uncorrelated and that the pollutants act independently in triggering asthma. The first assumption is reasonable in the case of these three pollutants. For example, a smoker’s home is no more or less likely to contain radon or mold than a nonsmoker’s home. On the other hand, the second assumption (biological independence) is problematic because evidence exists that these pollutants may act synergistically. For example, previous studies suggest that children exposed to both damp indoor environments (associated with mold growth) and environmental tobacco smoke have a greatly increased risk of developing asthma compared with the risk predicted by considering each of these exposures separately [15], [16]. Hence, our estimates of asthma risks associated with indoor air pollutants may be underestimates.

Combining the estimated *PAF* with the observed total number of cases of the health outcome (*D_total_*, from the fourth columns in Tables 1 and 2) gives the number of cases (deaths or health-care facility visits) attributable to the environmental exposure, *D_attrib_*, as shown in Equation 3.
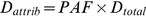
(3)


The relative risk columns in Tables 1 and 2 show the parameters for *RR(x)* in equation 1 (all based on previous international epidemiologic studies). Unless otherwise indicated, relative risks were characterized as lognormally distributed with the mean value and 95% confidence intervals drawn from the referenced studies. Relative risk estimates were selected by expert panels (Table S3) based on WHO guidance documents [17], [18] and relevant epidemiologic literature.

The “exposure estimation method” columns in Tables 1 and 2 show how we estimated the population distribution of exposure (*P(x)* in equation 1). Previously collected pollutant concentration data from EAD’s environmental monitoring networks were available for outdoor air and coastal water, and we collected new measurements of indoor air quality [19]. However, we had to impute occupational exposures based on local employment data and previous studies in other regions for similar industrial sectors and job descriptions; details are provided elsewhere [20]. Similarly, we had to impute exposure to drinking water contaminants based on local information about sources of drinking water and access to improved water and sanitation services, using methods established in previous global EBD studies [21].

Pollutant concentrations in outdoor air can vary highly even in relatively small sub-regions. To represent this variability, we subdivided the UAE into 55 km^2^ grid cells, each with different pollutant concentration parameters. Pollutant concentration parameters (mean and standard deviation) in each cell within the grid were estimated from a year’s worth of outdoor air monitoring data provided by EAD. Li et al. [14] provide additional details on the monitoring network and statistical estimation techniques.

Due to the complexities of representing variability and uncertainty, previous EBD estimates generally have represented the input variables in equations 1–3 as deterministic–that is, as variables having fixed values. However, this representation can be misleading because these variables may take on any of a variety of values, causing the actual EBD experienced by a specific population to differ considerably from predictions derived only from a single fixed value for each input. For example, the concentration, *x*, of a contaminant to which an individual is exposed varies with time and location and also may be uncertain due to limitations in pollutant monitoring systems. Similarly, the relative risks associated with exposure *RR(x)* also may vary by individual and be uncertain due to limitations in the epidemiologic studies from which they were estimated.

Morgan and Henrion [22], in their classic and widely recognized guide for incorporating technical and scientific uncertainty into risk analysis, observe Policies that ignore uncertainty about technology, and about the physical world, often lead in the long run to unsatisfactory technical, social, and political outcomes. By definition, risk involves an ‘exposure to a chance of injury or loss’ (Random House, 1966). The fact that risk inherently involves chance or probability leads directly to a need to describe and deal with uncertainty).

The EBD analysis presented here follows Morgan and Henrion’s recommended protocols for incorporating variability and uncertainty into risk analysis. We represented input variables that are uncertain and/or subject to variability as random variables (that is, as probability distributions). We then employed the Monte Carlo simulation technique to propagate the variability and uncertainty in input parameters through the calculations. In brief, equations 1–3 were computed thousands of times, each time using a new selection of input variable values drawn from the appropriate probability distributions; for a further explanation of this method, which is widely employed in risk assessment, see Morgan and Henrion [22] as well as Thomopoulos [23].

To implement the Monte Carlo simulation, we employed *Analytica* software (Lumina Decision Systems, Los Gatos, California), which was developed specifically to enable the modular construction of simulation models. Within *Analytica*, we specified appropriate probability distributions (or deterministic values where appropriate) for the input variables for each combination of contaminant and exposure pathway. We added nodes that carry out the computations represented by equations 1–3. *Analytica* then uses a method known as Latin hypercube sampling in order to compute a probability distribution for the results of equations 1–3 for each contaminant and exposure pathway based on samples drawn at random from the probability distributions for the input variables. For these estimations, we used 1,000 iterations (i.e., 1,000 samples of each input variable), which yielded stable results. The resulting estimates indicate not only the most likely number of diseases attributable to each contaminant-exposure pathway combination but also the range of plausible values, given the uncertainty and variability in existing information.

**Figure 1:**
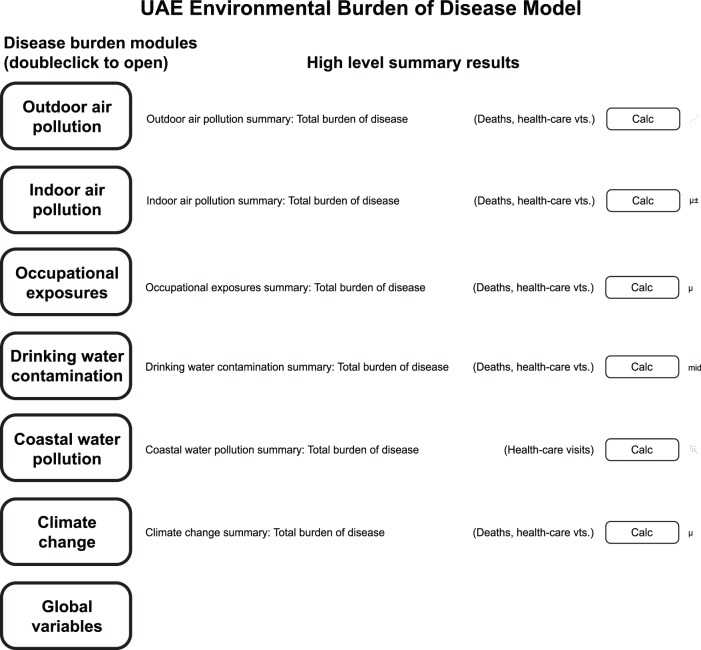
Top level of the UAE EBD model. Double-clicking on any node opens further layers of a module that shows how the EBD for each exposure route is estimated. The “global variables” node contains all health outcome and population distribution data for all modules.

**Figure 2:**
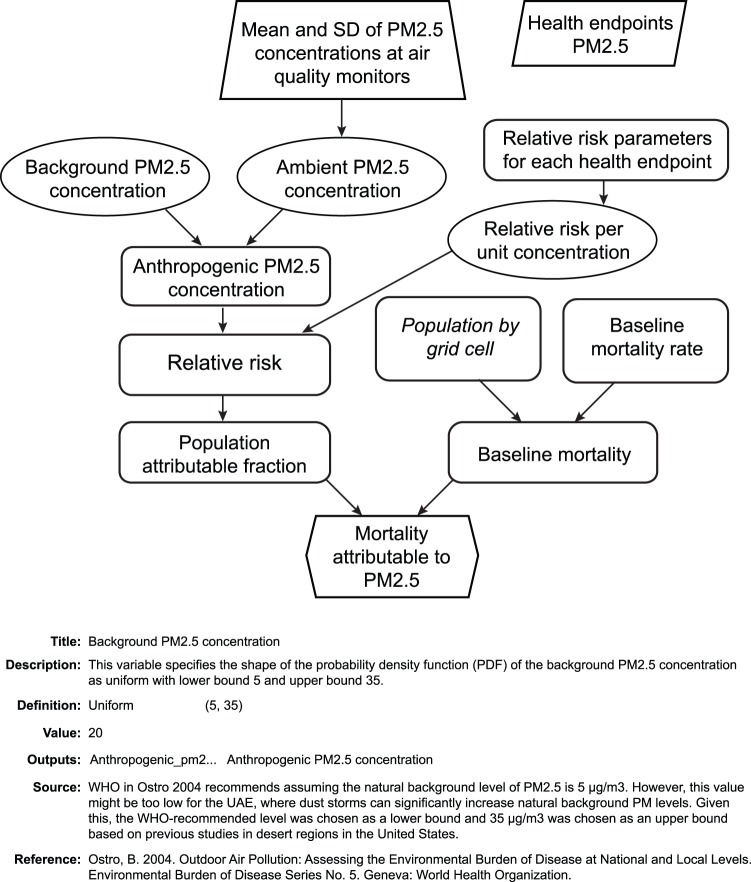
Layer within the model’s outdoor air pollution module and the notecard that opens when clicking on the “Background PM2.5 concentration” node. Trapezoids indicate deterministic variables; ovals indicate random variables; rectangles with rounded corners are variables determined from equations involving higher-level nodes; and the hexagon indicates an objective node. Key nodes are as follows: *Health endpoints PM2.5*: listing of health endpoints associated with PM_2.5_; *Background PM2.5 concentration*: PM_2.5_ concentration in the absence of human activity; *Mean and SD of PM2.5 concentrations at air quality monitors*: mean and SD of a year’s worth of measurements at UAE air quality monitors (interpolated from monitors for each of 1,164 cells in a grid used to divide the UAE into subunits for analysis); *Relative risk parameters for each health endpoint:* as shown in Table 1, last column; *Baseline mortality rate:* mortality rate by emirate and citizenship*; Population by grid cell:* population by citizenship in each of the 1,164 geographic grid cells.

Figure 1 shows the front page of the UAE EBD model. From within the *Analytica* program, double clicking on the name of an exposure route opens further layers, such as the layer illustrated in Figure 2. These lower layers further document the input variables and relationships among them. Figure 2 shows the bottom layer of the module for estimating the EBD due to PM_2.5_ in outdoor air. From within *Analytica*, double clicking on any variable icon opens a display that completely specifies the variable (e.g., observational data, parameters of the underlying probability distribution). An advantage of *Analytica* is its ability to handle very large matrix operations, which enabled us to divide the UAE into geographic subunits smaller than a single emirate for purposes of analysis when sufficient exposure data were available; each geographic subunit is represented as a row of a matrix, and population, baseline disease, and exposure information are assigned accordingly. A further advantage is that the visual construction facilitates communicating the methods to nonspecialists.

## Results

**Figure 3:**
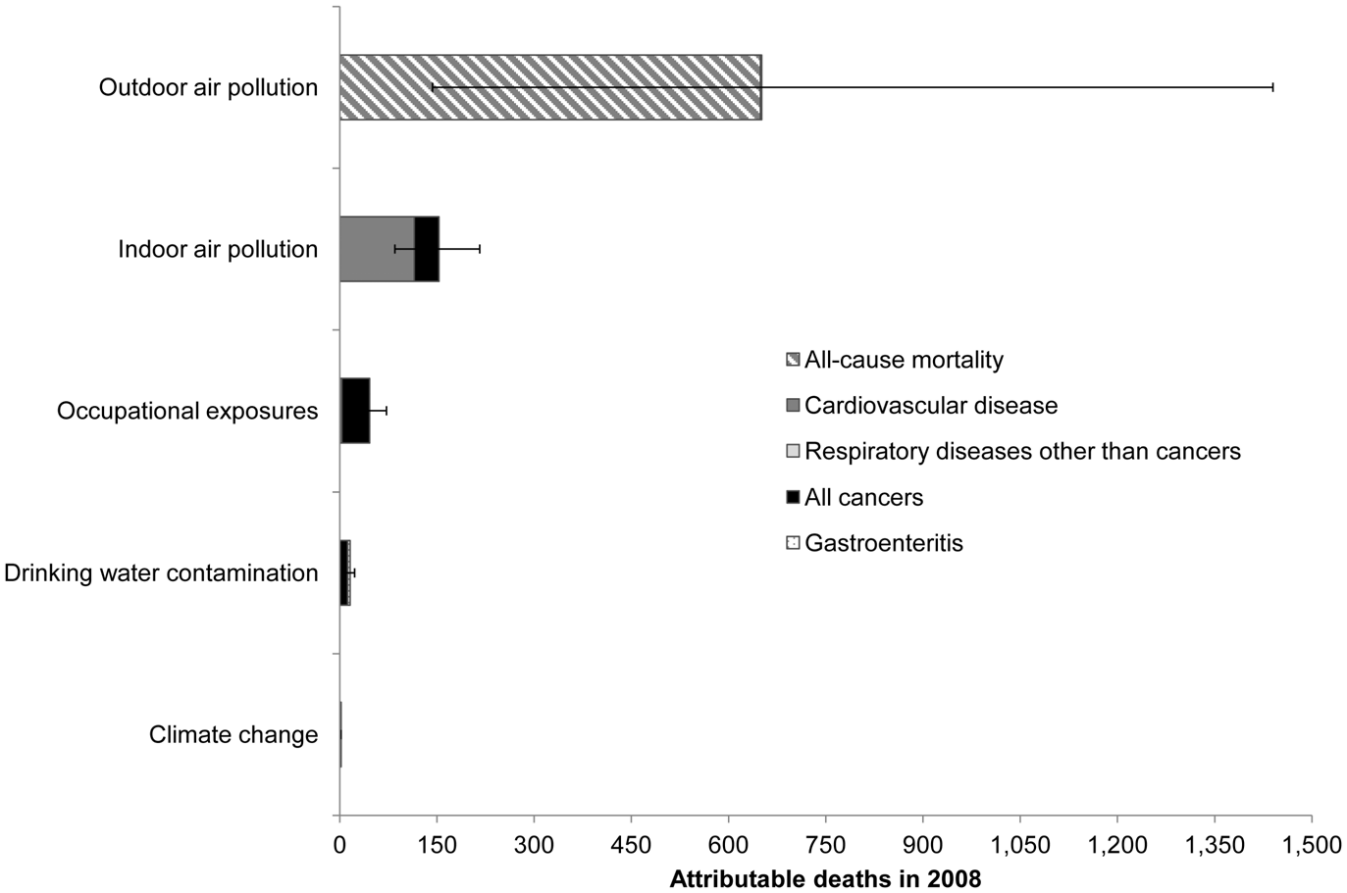
Estimated deaths attributable to environmental pollutants in the UAE. In addition to those easily seen in the chart, two deaths from respiratory diseases other than cancers were attributed to environmental pollution in 2008, both due to outdoor air pollution.

**Figure 4:**
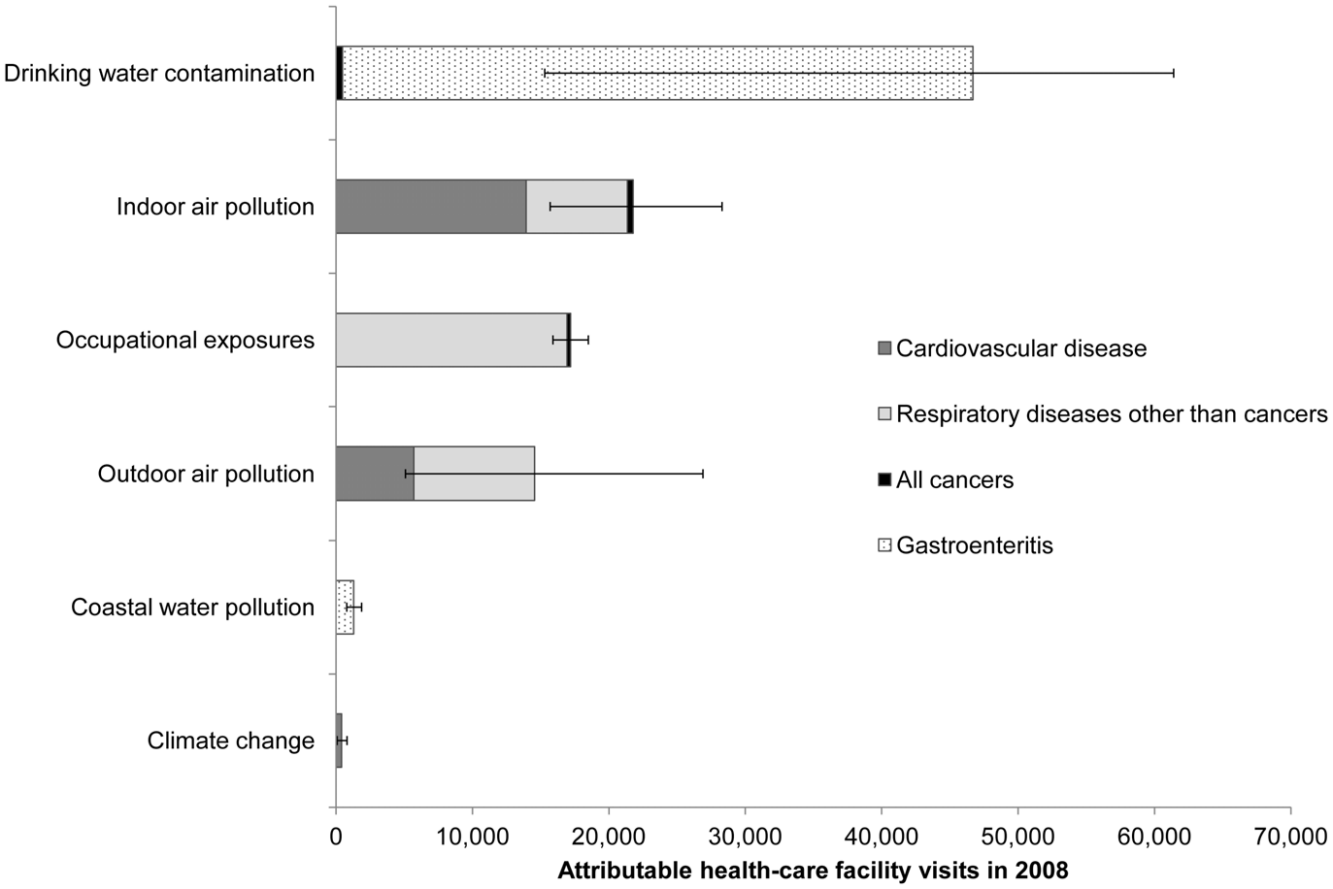
Estimated health-care facility visits attributable to environmental pollutants in the UAE.

Figures 3 and 4 illustrate the estimated deaths and health-care facility visits attributable to the environmental exposure routes considered in this analysis. Tables 3 and 4 provide detailed results, including the *PAF* for each exposure route–health endpoint combination.

**Table 3:** Deaths attributable to environmental pollution risk factors in 2008.

Exposure route	Cause of mortality	Attributable fraction	Attributable deaths	Confidence interval lower bound	Confidence interval upper bound
Outdoor air pollution	All causes (adults>30)	7.3%	649	143	1,438
	Respiratory disease (children<5)	7.4%	2	0	6
	Total		651	143	1,444
Indoor air pollution	Cardiovascular disease	5.0%	115	50	178
	Lung cancer	31.7%	38*	14	55
	Total		153	85	216
Occupational exposures	Asthma	10.0%	1	1	1
	Chronic obstructivepulmonary disease	5.4%	2	NA	NA
	Asbestosis	NA	0	NA	NA
	Silicosis	NA	0	NA	NA
	Malignant mesothelioma	100.0%	6	NA	NA
	Leukemia	9.2%	12	5	22
	Lung cancer	20.8%	25	12	41
	Total		46	26	72
Drinking water contamination	Bladder cancer	7.5%	3	1	5
	Colon cancer	10.0%	6	0	12
	Rectal cancer	57.1%	3	0	6
	Gastroenteritis	12.8%	4	1	5
	Total		15	8	23
Climate change	Cardiovascular disease	0.1%	2	0	2

* An additional two deaths (95% CI 1–3) may be attributable to radon exposure in Abu Dhabi city and Sharjah.

**Table 4:** Health-care facility visits attributable to environmental pollution risk factors in 2008.

Exposure route	Health outcome	Attributable fraction	Attributable health-care facility visits	Confidence interval lower bound	Confidence interval upper bound
Outdoor air pollution	Cardiovascular disease	1.9%	5,700	1,910	10,500
	Respiratory disease	5.0%	8,850	2,930	17,300
	Total		14,600	5,090	26,900
Indoor air pollution	Asthma (<18) (environmentaltobacco smoke and mold)	14.4%	3,510*	964	7,860
	Asthma (age≥18)	7.8%	2,541	63	4,730
	Cardiovascular disease	4.5%	13,940	9,620	18,200
	Lower respiratory tractinfection (age≤6)	9.7%	1,360	710	1,970
	Leukemia	19.0%	289	44	477
	Lung cancer	29.3%	130**	38	195
	Total		21,800	15,700	28,300
Occupational exposures	Asthma	16.5%	11,900	10,500	13,100
	Chronic obstructive pulmonarydisease	18.4%	5,010		
	Asbestosis	100.0%	3		
	Silicosis	100.0%	8		
	Malignant mesothelioma	89.3%	25		
	Leukemia	9.1%	138	57	255
	Lung cancer	25.3%	112	54	180
	Total		17,200	15,900	18,500
Drinking water contamination	Bladder cancer	16.6%	154	10	296
	Colon cancer	10.6%	232	0	569
	Rectal cancer	15.0%	96	0	219
	Gastroenteritis	57.0%	46,200	14,700	60,900
	Total		46,600	15,300	61,400
Coastal water pollution	Gastroenteritis	1.6%	1,300	792	1,880
Climate change	Cardiovascular disease	0.2%	410	84	802

* Formaldehyde exposure adds another 74 (95% confidence interval 50–99) visits for children under age 6, not included in this total. The total here accounts for the combined risks of ETS and mold exposure and assumes those risks are independent.

** An additional 20 visits (95% confidence interval 12–28) may occur due to radon exposure in Abu Dhabi city and Sharjah.

As Figure 3 indicates, outdoor air pollution contributed to 651 deaths (95% CI 143–1,440) in 2008–more than all the other risk factors combined. Indoor air pollution and occupational exposures were the second and third leading contributors to mortality, with 153 (95% CI 85–216) and 46 deaths (95% CI 26–72) attributable to these factors, respectively.

The estimated health-care facility visits attributable to environmental pollution show a different prioritization (Figure 4). Drinking water pollution was the leading contributor, with an estimated 46,600 (95% CI 15,300–61,400) attributable visits (approximately 15% of the health-care facility visits for all the illnesses considered in this study). Indoor air pollution, occupational exposures, and outdoor air pollution contributed to similar numbers of visits (15,000–20,000 each). While these results present a somewhat different ordering than those shown for deaths in Figure 3, it is important to note that the severity of the illnesses prompting these health-care facility visits varies considerably. For example, a heart attack (to which indoor and outdoor air pollution are important risk contributors) is much more severe than a case of mild gastroenteritis associated with drinking water contamination, especially in a wealthy nation such as the UAE, where child mortality is very low and medical assistance is readily available. Previous EBD studies have typically reported results as disability-adjusted life years (DALYs), representing the number of healthy life years lost due to living in a state of less than perfect health or dying prematurely [17], but the data necessary to translate health-care facility visits into DALYs were unavailable in this case. Future studies using DALYs as a unit of measure would account for such differences and provide a single prioritized ranking.

**Figure 5:**
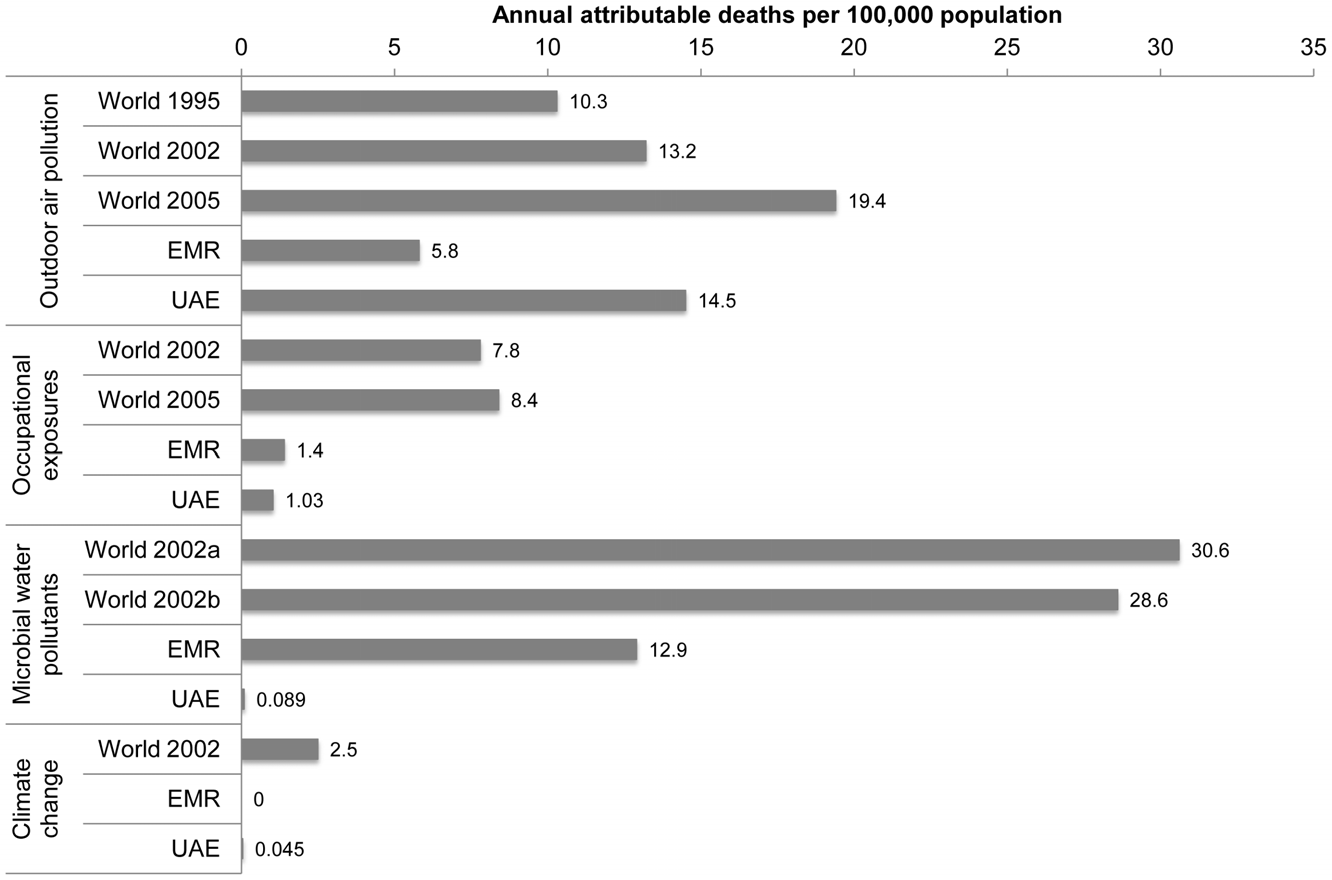
Comparison of annual deaths attributed to environmental pollutants in the UAE with previous global and regional estimates. See Table S2 for sources of international comparison estimates.

Figure 5 compares our estimates of the per-capita attributable deaths to previous global estimates and the WHO’s estimates for the Eastern Mediterranean Region for environmental risk factors for which comparable information is available. As shown, the UAE’s environmental burden of disease is low for all the factors for which relevant comparisons were available except for outdoor air pollution (see Table S2 for additional details on previous studies).

## Discussion

These results provide an initial indication of the public health benefits possible in the UAE through new interventions to reduce environmental pollution. Reducing pollutant concentrations in outdoor and indoor air and protecting workers from occupational pollutant exposures could prevent hundreds of premature deaths and tens of thousands of health-care facility visits each year. In addition, although the risk of gastrointestinal illnesses due to water pollution is very low in the UAE by global standards, additional measures to improve drinking water and coastal water quality could prevent a large fraction of mild gastrointestinal illnesses.

In many developed countries, a fear of cancer has driven programs to reduce environmental pollution [24]. It is interesting to note that environmental pollution is associated with a far larger number of deaths for noncancer health endpoints, especially cardiovascular disease, which is the leading cause of death in the UAE.

**Figure 6:**
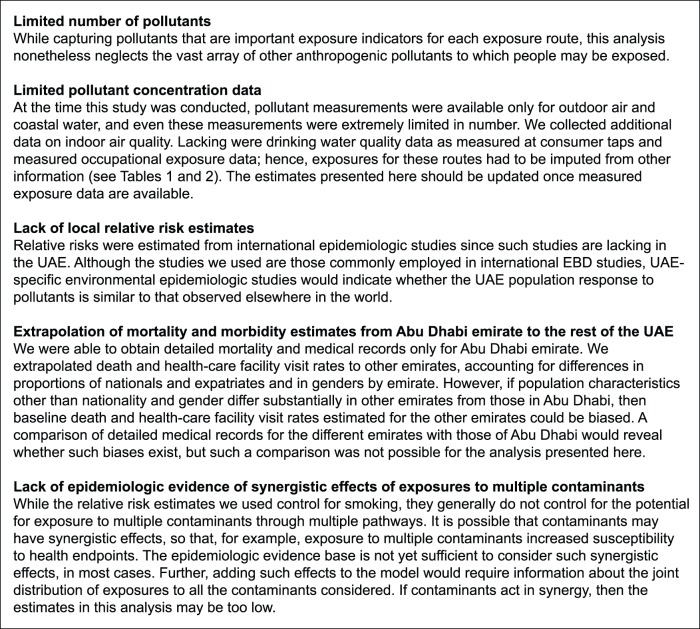
Key sources of uncertainty.

These results provide one indication of the relative impacts environmental pollutants on health in the UAE and should not be considered as exact. Indeed, the large confidence intervals (Figures 3 and 4) indicate a high level of uncertainty. Figure 6 lists important sources of uncertainty.

In addition to these uncertainties, a further limitation of the study is the inability to combine deaths and health-care facility visits into a single summary measure, such as DALYs, which would facilitate comparisons among risk factors. The information needed to calculate DALYs for the UAE population was unavailable for this study. Most importantly, calculating DALYs requires that each illness experienced by a member of the population is counted only once. However, the data available to us listed each visit to a health-care facility regardless of whether the visit was a repeat encounter for a previously diagnosed illness. To protect patient confidentiality and due to the evolving nature of the UAE’s medical record systems, we were not provided with information that would have enabled us to screen out these multiple visits. Hence, one respiratory illness case would be counted four times in the database if the same patient returned to the doctor three times after the initial visit. Hence, had we attempted to calculate DALYs, the results would have been inflated due to such repeat visits. Although it was not possible to compute DALYs, the indicators used in this research (deaths and health-care facility visits) nonetheless provide valuable information about the relative impacts of different types of environmental pollution in the UAE from a public health perspective.

Despite these limitations, this study builds on methods that the WHO has advocated over the past decade in order to support national efforts to reduce environmental risks to health [6], [18]. The results yield information about opportunities to prevent illnesses and reduce health-care spending through new environmental interventions. Indeed, in part as a result of this study, Abu Dhabi is already pursuing a number of new programs to reduce environmental pollution exposures, including upgrading its outdoor air monitoring system in order to plan for air quality improvements, establishing a new occupational health management system, implementing an aggressive marine water quality monitoring program, and instituting new anti-smoking campaigns.

## Supporting Information

Figure S1 **The UAE comprises seven emirates.**
(EPS)

Table S1 Population distribution of the UAE by emirate and gender (2008).
(DOCX)

Table S2 Previous estimates of deaths attributable to environmental pollution.
(DOCX)

Table S3 Experts contributing to burden of disease analyses for each exposure route.
(DOCX)
