# Supplementary material for: Deaths and Medical Visits Attributable to Environmental Pollution in the United Arab Emirates
Source: PLoS One. 2013 Mar 4;8(3):e57536. doi: 10.1371/journal.pone.0057536 (PMC3587618; doi:10.1371/journal.pone.0057536)
Supplement: Table S1 — Population distribution of the UAE by emirate and gender (2008). (DOCX) [file pone.0057536.s002.docx]

**Table S1. Population distribution of the UAE by emirate and gender (2008)**

|  | **Citizen Population** | | **Noncitizen Population** | | **Total** |
| --- | --- | --- | --- | --- | --- |
| **Emirate** | **Male** | **Female** | **Male** | **Female** |  |
| Abu Dhabi | 188,000 | 184,000 | 794,000 | 327,000 | 1,493,000 |
| Dubai | 71,000 | 70,000 | 1,050,000 | 287,000 | 1,478,000 |
| Sharjah | 74,000 | 69,000 | 507,000 | 232,000 | 882,000 |
| Ajman | 21,000 | 20,000 | 123,000 | 60,000 | 224,000 |
| Umm Al Quwain | 8,000 | 8,000 | 24,000 | 12,000 | 52,000 |
| Ras Al Khaimah | 46,000 | 45,000 | 92,000 | 39,000 | 222,000 |
| Fujairah | 30,000 | 30,000 | 56,000 | 21,000 | 127,000 |
| Total | 438,000 | 426,000 | 2,646,000 | 978,000 | 4,488,000 |
