# Supplementary material for: Deaths and Medical Visits Attributable to Environmental Pollution in the United Arab Emirates
Source: PLoS One. 2013 Mar 4;8(3):e57536. doi: 10.1371/journal.pone.0057536 (PMC3587618; doi:10.1371/journal.pone.0057536)
Supplement: Table S2 — Previous estimates of deaths attributable to environmental pollution. (DOCX) [file pone.0057536.s003.docx]

**Table S2. Previous estimates of deaths attributable to environmental pollution**

| **Exposure pathway** | **Pollutant(s)** | **Study population** | **Year of publication** | **Attributable deaths** | **% of deaths from all causes** | **Number per 10,000 people** |
| --- | --- | --- | --- | --- | --- | --- |
| Outdoor air | Total suspended particles and SO_2_ | World [46, 47] | 1995 | 500,000 | 1.06% | 1.03 |
|  | Particulate matter (PM) | China [48] | 1997 | 153,500 | 1.82% | 1.24 |
|  | PM_10_ and PM_2.5_ | World [49] | 2002 | 799,000 | 1.43% | 1.32 |
|  | PM_10_ and PM_2.5_ | EMR countries with low child and adult mortality [49] | 2002 | 8,000 | 1.15% | 0.58 |
|  | PM_10_ | European children < 5 [50] | 2004 | 13,796 | 6.40% | 2.68 |
|  | PM_2.5_ | World [51] | 2005 | 799,000 | 1.36% | 1.24 |
| Indoor air | Emissions from solid fuel combustion | World and PM_2.5_ [47, 52] | 1997 | 2,800,000 | 5.94% | 5.76 |
|  | Emissions from solid fuel combustion | World [47, 52] | 1997 | 2,200,000 | 4.67% | 4.53 |
|  | PM and emissions from solid fuel combustion | China [48] | 1997 | 960,000 | 11.39% | 7.75 |
|  | Emissions from solid fuel combustion | India: Children under 5 and adult women [47] | 2000 | 424,000 | 11.86% | 10.67 |
|  | Emissions from solid fuel combustion | China: Children under 5 and adult women [47] | 2000 | 423,000 | 12.27% | 8.47 |
|  | Emissions from solid fuel combustion | World [49] | 2002 | 1,619,000 | 2.90% | 2.68 |
|  | Emissions from solid fuel combustion | EMR countries with low child and adult mortality [49] | 2002 | 2,000 | 0.29% | 0.14 |
|  | Emissions from solid fuel combustion | World: Children under 5 and adult women [47] | 2003 | 1,600,000 | 7.52% | 6.47 |
|  | Emissions from solid fuel combustion | European children < 5 [50] | 2004 | 9,845 | 4.57% | 1.91 |
|  | Emissions from solid fuel combustion | Indonesian children < 5 and women > 30 [53] | 2009 | 14,000 | 2.73% | 2.01 |
|  | Emissions from solid fuel combustion | Philippino children < 5 and all population > 15 [53] | 2009 | 5,700 | 1.58% | 0.86 |
|  | Emissions from solid fuel combustion | Timor-Leste children < 5 and women > 30 [53] | 2009 | 305 | 12.84% | 10.57 |
| Occupational exposures | Carcinogens and airborne particulates | EMR countries with low child and adult mortality [49] | 2002 | 2,000 | 0.29% | 0.14 |
|  | Carcinogens and airborne particulates | World [49] | 2002 | 474,000 | 0.85% | 0.78 |
|  | Chronic obstructive pulmonary disease, occupational carcinogens, and respiratory irritants | World [54] | 2005 | 538,000 | 0.92% | 0.84 |
| Water, sanitation, and hygiene (excluding recreational exposures) | Microbial pathogens | World [55] | 2002 | 2,187,000 | 3.92% | 3.62 |
|  | Microbial pathogens | World [49] | 2002 | 1,730,000 | 3.10% | 2.86 |
|  | Microbial pathogens | EMR, countries with low child and adult mortality [49] | 2002 | 18,000 | 2.59% | 1.29 |
|  | Microbial pathogens | European children < 15 [56] | 2004 | 13,548 | 5.30% | 0.78 |
| Climate change | Future climate scenarios based on various carbon emissions and concentrations | World [49] | 2002 | 154,000 | 0.28% | 0.25 |
|  | Future climate scenarios based on various carbon emissions and concentrations | EMR countries with low child and adult mortality [49] | 2002 | 0 | 0.00% | 0.00 |

46 Hong C (1995) Global burden of disease from air pollution. Geneva: World Health Organization.

47 Smith KR, Mehta S (2003) The burden of disease from indoor air pollution in developing countries: Comparison of estimates. Int J Hyg Environ Health 206(4-5): 279-289.

48 Florig HK (1997) China’s air pollution risks. Environ Sci Technol 31(6): 4-9.

49 Ezzati M, Lopez AD, Rodgers A, Van der Hoorn S, Murray CJL (2002) Comparative risk assessment collaborative group: Selected major risk factors and global and regional burden of disease. Lancet 360: 1347-1360.

50 Valent F, Little DA, Bertollini R, Nemer LE, Barbone F, et al. (2004) Burden of disease attributable to selected environmental factors and injury among children and adolescents in Europe. Lancet 363: 2032-2039.

51 Cohen AJ, Ross Anderson H, Ostro B, Pandey KD, Krzyzanowski M, et al. (2005) The global burden of disease due to outdoor air pollution. J Toxicol Environ Health A 68(13-14): 1301-1307.

52 World Health Organization (1997) Health and environment in sustainable development. Geneva: World Health Organization.

53 Arcenas A, Bojö J, Larsen B, Ruiz Ñunez F (2010) The economic costs of indoor air pollution: New results for Indonesia, the Philippines, and Timor-Leste. J Nat Res Pol Res 2(1): 75-93.

54 Nelson D, Concha-Barrientos M, Driscoll T, Steenland K, Fingerhut M, et al. (2005) The global burden of selected occupational diseases and injury risks: Methodology and summary. Am J Ind Med 418: 400-418.

55 Prüss A, Kay D, Fewtrell L, Bartram J (2002) Estimating the burden of disease from water, sanitation, and hygiene at a global level. Environ Health Perspect 110(5): 537-542.

56 Valent F, Little DA (2004) Environmental burden of disease series, No. 8: Burden of disease attributable to selected environmental factors and injuries among Europe’s children and adolescents. Geneva: World Health Organization.
