# Supplementary material for: Deaths and Medical Visits Attributable to Environmental Pollution in the United Arab Emirates
Source: PLoS One. 2013 Mar 4;8(3):e57536. doi: 10.1371/journal.pone.0057536 (PMC3587618; doi:10.1371/journal.pone.0057536)
Supplement: Table S3 — Experts contributing to burden of disease analyses for each exposure route. (DOCX) [file pone.0057536.s004.docx]

**Table S3. Experts contributing to burden of disease analyses for each exposure route**

| **Exposure route** | **Expert contributor** |
| --- | --- |
| Outdoor air | Dr. Ying Li, Dr. Gavino Puggioni, Mr. Prahlad Jat, Dr. Marc Serre, Dr. Kenneth G. Sexton, Dr. J. Jason West, Dr. Saravanan Arunachalam, Ms. Uma Shankar, Dr. William Vizuete, Dr. Mohammed Zuber Farooqui |
| Indoor air | Ms. Chris B. Trent, Dr. David Leith, Dr. Michael Flynn |
| Occupational exposures | Dr. Leena A. Nylander-French, Ms. Tiina Folley |
| Climate change | Dr. J. Jason West, Dr. Richard N. L. Andrews, Ms. Leslie Chinery, |
| Drinking water contamination | Dr. Gregory Characklis, Dr. Joseph N. LoBuglio |
| Coastal water pollution | Dr. Gregory Characklis, Dr. Leigh-Anne H. Krometis, Dr. Joseph N. LoBuglio |

Note: At the time this analysis was conducted, all experts listed above were affiliated with the Gillings School of Global Public Health at the University of North Carolina–Chapel Hill.
